# Effect of partial mutton meat substitution with Bambara groundnut (*Vigna subterranea* (L.) Verdc.) flour on physicochemical properties, lipid oxidation, and sensory acceptability of low‐fat patties

**DOI:** 10.1002/fsn3.4059

**Published:** 2024-03-04

**Authors:** Kgaogelo Edwin Ramatsetse, Shonisani Eugenia Ramashia, Mpho Edward Mashau

**Affiliations:** ^1^ Department of Food Science and Technology, Faculty of Science, Engineering and Agriculture University of Venda Thohoyandou South Africa; ^2^ School of Bioengineering and Food Technology, Faculty of Applied Sciences and Biotechnology Shoolini University Solan Himachal Pradesh India

**Keywords:** Bambara groundnut, lipid oxidation, mutton patties, physicochemical properties, sensory acceptability

## Abstract

Health concerns regarding fat consumption, as well as shifts in customer preference, have prompted substantial studies into low‐fat products. This study examined the nutritional, color, functional, and antioxidant properties of Bambara groundnut (BGN) flour varieties (cream, brown, and red‐coated) grains as well as their influence on the physicochemical properties, lipid oxidation, and sensory acceptability of low‐fat mutton patties. The patties were formulated with 2.5%, 5%, 7.5%, and 10% of BGN flour for each variety, and 100% mutton patties were used as a control. The BGN flours showed significant (*p* < .05) differences in their nutritional composition (except for ash content), color, functional (excluding emulsion stability), and antioxidant properties. The increase in the percentage of substitution of BGN flours significantly increased the fiber (0.00% to 0.79%), ash (1.16*%* to 1.99%), and carbohydrates (2.14*%* to 1.99%) contents of the formulated mutton patties. However, moisture and protein contents decreased. The cooking yield of the formulated patties significantly increased with the increase in the percentage substitution of BGN flours (2.5%–10%), with values ranging from 76.39*%* to 86.80%, but the diameter reduction was limited. The increase in the inclusion of BGN flours significantly increased the lightness, hue angle, color difference, and whiteness of patties. Nevertheless, the redness, yellowness, chroma, and yellowness index of the patties decreased. The hardness and resilience of formulated patties significantly increased, with values varying from 16.41 to 17.66 N, and from 0.35 to 0.48 J/J, respectively, whereas the springiness, cohesiveness, and chewiness decreased. The lipid oxidation of formulated mutton patties significantly increased from Days 7 to 21, but was still less than that of the control sample within storage days. The sensory properties of formulated patties were not significantly different from the control sample and were above the acceptable score of five. All BGN varieties had positive effects on the mutton patties, more especially red, followed by brown, and cream, respectively. The inclusion of a 10% red BGN flour variety is highly recommended due to its significant impact on mutton patties. Nevertheless, different types of BGN varieties can each be used as additives in mutton patties without having detrimental effects on the quality parameters of the patties.

## Graphical abstract

Physicochemical properties, lipid oxidation, and sensory acceptability of low‐fat mutton patties substituted with Bambara groundnut flour were determined. The increase in the percentage substitution of BGN flours significantly increased the fiber, ash, and carbohydrate contents of the formulated mutton patties. The cooking yield of the formulated patties significantly increased with the increase in the percentage substitution of BGN flours. The hardness and resilience of formulated patties significantly increased.

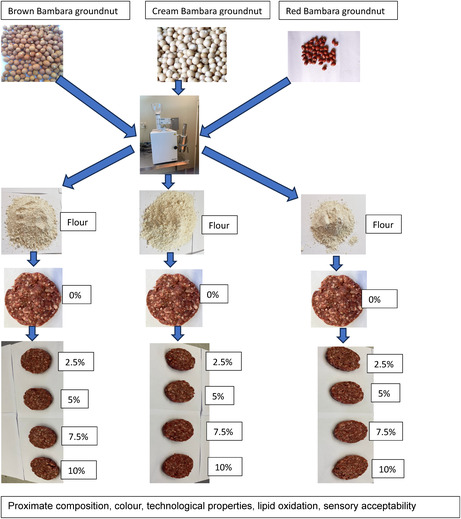

## 1 INTRODUCTION

Meat is a nutrient‐dense food made up of water, proteins, lipids, vitamins, and minerals, as well as small amount of carbohydrates (Aminzare et al., 2019). Mutton meat is from an adult sheep, and it is not tender. Mutton is India's most common meat, owing to its lack of social and religious restrictions (Mendiratta et al., 2013). This is the primary reason why mutton meat is still more expensive than other meats. Nevertheless, consuming a lot of processed meat has negative effects, such as causing cardiovascular disease or colon cancer (Grosso et al., 2022; Willett et al., 2019). Non‐meat additives such as soybeans, eggs, durum wheat powder, potato mash, rice, and milk products are widely used as extenders, binders, and fillers in minced meat products to increase quality features and minimize production losses (Possidonio et al., 2021; Yang, 2019).

Consumers are currently very concerned with their well‐being and the food products they consume. Therefore, the meat industry must process meat that is lean, less fat, and rich in protein to meet the claim of nourishing and balancing the diet. Health concerns regarding fat consumption, as well as shifts in customer liking, have prompted substantial studies into low‐fat products (Di Vita et al., 2022; Yogesh, 2021). The addition of legumes as non‐meat additives in manufactured meat products is a potential answer to the current market trend for low‐fat and high‐fiber meat products (Ponnampalam et al., 2019). For example, Bambara groundnut (*Vigna subterranea* (L.) Verdc.) contains a 5.8% level of crude fat, and it can be categorized as a source of healthy fat that can be employed in less‐fat diet formulations such as lean meat (Oyeleke et al., 2012). Besides their intrinsic functional properties, the incorporation of legumes, fruits, and vegetables into comminuted meat products increases yield, oxidative stability, nutritional composition, and fiber content and reduces production costs (Beriain et al., 2018; Kumar et al., 2016).

There is significant information that the consumption of legumes, including Bambara groundnut (BGN) and other crop products, is good for human well‐being and decreases the chances of being exposed to cardiovascular diseases (Schwingshackl et al., 2018; Ramatsetse et al., 2023). The antioxidant properties of BGN can prevent non‐transmissible diseases such as diabetes, malignancy, Alzheimer, and arthritis (Ramatsetse et al., 2023). Moreover, rising consumers' desire for healthier food products is spurring the production of novel products that are more sustainable, such as incorporating plant sources such as BGN (Rout et al., 2022). Mubaiwa et al. (2018) state that the high amount of protein in BGN provides the spotlight more on creating updated processes for its production and extending its utilization. The varied nutritional component of BGN grains implies that they have the capacity to fulfil the nutritional demands of a significant number of people.

Meat fat serves as a flavor compound reservoir and contributes to food texture. As a result, moisture drip and fat may affect or change product quality (Alves et al., 2016). The loss of liquid decreases the sensory result, the heaviness of the commodity, and possibly its selling value. Shrinkage may also be caused by a lack of fluid in the product. Utilizing plant‐based protein derivatives can help to lessen the moisture loss that takes place when cooking (Aslinah et al., 2018; Ravani & Sharma, 2022). Different non‐meat additives have been used in processed meat products. For instance, Bagdatli (2018) added quinoa flour to beef meatballs, Amadi (2020) added soybean to buffalo meat patties, and Ayandipe et al. (2020) added cassava and coconut composite powder to chicken sausages. The introduction of non‐meat ingredients such as quinoa and BGN flours in processed meat products improves sensory properties such as chewiness, juiciness, and taste (Alakali et al., 2010; Muchekeza et al., 2021).

There is little data regarding the utilization of BGN flour in processed meat products. Alakali et al. (2010) investigated the impact of BGN flour on the nutritional qualities and sensory properties of beef patties stored at 4°C for 21 days. The physicochemical, microbiological, and sensory properties of the formulated patties were acceptable over 21 days of refrigeration. Nevertheless, consumers preferred formulated beef patties added with up to 5% BGN flour. Dzudie et al. (2002) state that BGN flour was used because it is a cheaper source of protein and improves water retention and the production of structure in processed meat products. The limited use of BGN, like with other pulses, is associated with several problems, including the hard‐to‐cook and hard‐to‐mill characteristics of the grains, which also affect the nutritional quality (Diedericks et al., 2020; Gwala et al., 2019). To overcome these problems, the BGN grains are processed into flour. The incorporation of BGN flour in different foods could improve its utilization, especially in developing countries. To fully explore the use of BGN flours in meat products, it is crucial to assess their effect on the physicochemical, lipid oxidation, and sensory acceptability of low‐fat meat products. Therefore, this study assessed the nutritional, color, functional, and antioxidant properties of BGN flour varieties (cream, brown, and red coated grains) as well as their influence on the physicochemical properties, lipid oxidation, and sensory acceptability of mutton patties. The novelty of this research arises from the utilization of cream, brown, and red BGN flours in the development of low‐fat mutton patties and reporting the results for the very first time. Our hypothesis is that BGN flours will improve the nutritional value, technological properties, and sensory characteristics of mutton patties. The results of this study will promote the utilization of BGN flours in food products.

## 2 MATERIALS AND METHODS

### 2.1 Raw materials, chemicals, and reagents

Ten kilograms of mixed BGN (brown, cream, and red‐coated) grains were purchased from a supermarket in Thohoyandou, Limpopo Province, South Africa. The reagents acetone, acetic acid, methanol, ethanol, Folin–Ciocalteau, hydrochloricacid, and thiorbarbituric acid were purchased from Merck (Pty, Ltd., Midrand, Gauteng province, South Africa). All the chemicals used were of analytical grade.

### 2.2 Preparation of Bambara groundnut flour

The BGN grains were grouped according to their color (brown, cream, and red) separately and washed properly with tap water to remove dirt. Small branches and premature grains were physically discarded. The grains were then oven‐dried at 37°C for 48 h. The dried grains were ground using a Retsch miller (ultra‐centrifugal mill ZM 200, Germany) and sieved using a 212 μm sieve size to produce the final finer powder, packaged inside vacuum plastic bags, and stored in a dry, cool area until utilization.

### 2.3 Preparation of mutton patties

Ten kilograms of deboned mutton meat were bought at the Newco meat butchery in Shayandima, Limpopo Province, South Africa. Every ounce of subcutaneous and intramuscular fat as well as connective tissues were manually trimmed off, and then the meat was chopped into small pieces and ground using a meat mincer (P‐22, Tallers Ramon, Barcelona, Spain) through 5 mm plates. Throughout the mincing process, cold water with ice was added. Four blends (for each BGN variety) were formulated by combining minced mutton meat with 2.5%, 5.0%, 7.5%, and 10% of BGN flours, and 100% mutton patties were used as a control. To get a uniform mixture, each part was quietly mixed manually in a container for approximately 5 min. A petridish was used to shape the mix to form the patties (75 mm × 15 mm). Two batches were produced and packaged in low‐density polyethylene bags. For analysis of lipid oxidation, the patties were stored in the refrigerator at 4 ± 2°C and analyzed at Days 0, 7, 14, and 21. The experiment and analyses were duplicated to validate the results.

### 2.4 Determination of functional properties of Bambara groundnut flour

#### 2.4.1 Water absorption capacity and oil absorption capacity

Water absorption capacity (WAC) and oil absorption capacity (OAC) were obtained in triplicate, where one gram of sample was measured into 25 mL centrifuge tubes. A small amount of distilled and refined sunflower oil was added and stirred till the samples became separated with water or oil. The flours were centrifuged for 20 min at 3000 rpm and allowed to rest for 30 min at ambient conditions. The water or oil that was released as a result of centrifugation was emptied (Ferreira et al., 2018). The OAC and WAC were calculated as follows:

(1) $$\text{Water absorption capacity}\;\left(\%\right)=\frac{\text{weight of water absorbed}}{\text{weight of the sample}}\times 100$$

(2) $$\text{Oil absorption capacity}\;\left(\%\right)=\frac{\text{weight of oil absorbed}}{\text{weight of the sample}}\times 100$$

#### 2.4.2 Emulsifying activity

Emulsifying activity was determined following the procedure outlined by Arise et al. (2015). One gram of flour was put in 15 mL of water in centrifuge tubes and centrifuged at 20,000 rpm for 30 s for homogenization; then sunflower oil (15 mL) was poured, emulsified for 1.5 min, and centrifuged at 750 rpm for 5 min. Emulsifying activity was computed using the formula:

(3) $$\text{Emulsifying activity}\;\left(\%\right)=\frac{\text{Height of the remaining emuslfied layer}}{\text{Height of the whole tube content}}\times 100$$

#### 2.4.3 Emulsion stability

Emulsion stability was measured similarly to the emulsifying activity, but with the flour being heated at 80°C for 30 min, then cooled in tap water for 15 min, and centrifuged at 750 rpm for 5 min. Emulsion stability was computed using the formula:

(4) $$\text{Emulsion stability}\;\left(\%\right)=\frac{\text{Height of the remaing emulsifying layer}}{\text{Height of the whole tube content}}\times 100$$

#### 2.4.4 Bulk density

Bulk density was measured using the procedure outlined by Jenfa and Akinrinde (2019). Twenty grams of the powder put in a 100 mL measuring cylinder and hit at the base with a hand. The ultimate volume was measured after the cylinder was put on a flat surface. The bulk density was determined using the formula below:

(5) $$\text{Bulk density}=\frac{\text{Weight of sample}\;g/\mathrm{mL}}{\text{Volume of the sample}}$$

#### 2.4.5 Swelling capacity

Swelling capacity was determined using the procedure explained by Arise et al. (2019). Ten grams of BGN flour were put in a 300‐mL measuring cylinder, and the volume covered was measured. The powder was then poured into about 150 mL of distilled water and set aside for 4 h. Following swelling, the ultimate volume was measured. The swelling percentage was determined using the formula:

(6) $$\text{Swelling capacity}\;\left(\%\right)=\frac{\text{Final volume}-\text{Initial volume}}{\text{Initial volume}}\times 100$$

#### 2.4.6 Dispensability

Dispensability was determined using a procedure outlined by Jenfa and Akinrinde (2019). In a 100 mL measuring cylinder, 10 g of BGN flour was added, and 100 mL of distilled water was then poured. The mixture was aggressively agitated and left to rest for 3 h, then the volume of settled particles was recorded. The dispensability was determined using the following formula:

(7) $$\text{Dispensability}\;\left(\%\right)=100–\text{The volume of settled particles}$$

#### 2.4.7 Viscosity

The viscosity of the sample was obtained using the Brookfield viscometer (RUVDE230, USA). About 3.5 g of the sample (adjusted to 14% moisture content) and distilled water were poured to get a mass of the sample of about 25 g. The sample was heated at 50°C for 1 min while stirring to enable complete dispersion. The resistance of the flour mixture to the mixing paddles of the viscometer was used to measure the viscosity (cP) (Mudau et al., 2022).

### 2.5 Determination of antioxidant properties of Bambara groundnut flour

#### 2.5.1 DPPH radical‐scavenging activity of mutton patties

The DPPH assay was determined using a procedure outlined by Ofosu et al. (2020) with slight modifications. Two grams of each flour were measured and placed inside beakers, and 20 mL of methanol (30%) was poured and sornicated for 10 min in an ultrasonic bath. Afterwards, the samples were then centrifuged (Rotina 380R‐Labotec Ecotherm, Midrand, South Africa) at 3000 rpm for 10 min and filtered into beakers utilizing Whatman filter paper. Two millimeters of the sample extract were added to 2 mL of 0.1 mm DPPH in 10 mL test tubes. The mixture was shaken vigorously, kept at ambient conditions for 30 min, and absorbance was analyzed at 517 nm using a UV‐spectrophotometer (Shimadzu UV‐1800, Japan). The DPPH content was computed using the following equation:

(8) $$\text{DPPH}\;\left(\%\right)=\frac{\text{Absorbance of the control}\;\left(\text{methanol with DPPH solution}\right)-\text{Absorbance of the flour sample}}{\text{Absorbance of the control}\;\left(\text{methanol with DPPH solution}\right)}\times 100$$

#### 2.5.2 Total phenolic content

Total phenolic content was analyzed using a procedure outlined by Mahmoud et al. (2017) with slight modifications. Two grams of each flour were measured and deposited into the beakers, and 20 mL of methanol (30%) was then poured and sornicated for 10 min in an ultrasonic bath. The mixture was centrifuged at 3000 rpm for 10 min and filtered into beakers. Thereafter, 0.5 mL of the sample was poured into test tubes, and 1.5 mL of Folin–Ciocalteau reagent was also poured and let stand for 5 min at ambient conditions (25 ± 2°C). After 5 min, 2 mL of sodium carbonate (7%) was poured and placed in an incubator for 50 min in a dim place with intermittent shaking. The absorbance of the samples was determined using a UV‐spectrophotometer (Shimadzu UV‐1800, Japan) at 725 nm. The total phenolic content was computed using the standard curve and reported as gallic acid (mg GAE/g).

#### 2.5.3 Flavonoids content

Total flavonoid content was analyzed using a colorimetric procedure. Two grams of each flour were measured and placed into beakers, and 20 mL of methanol (30%) was then poured and sornicated for 10 min in an ultrasonic bath. Afterwards, the mixture was centrifuged at 5000 rpm for 10 min and filtered using Whatman paper. Then, 0.3 mL of sample extracts was added into a test tube, 3.4 mL of 30% methanol was added, and 0.15 mL of sodium nitrite (NaNo_2_) was also added. Thereafter, 0.15 mL of aluminum chloride hexahydrate (AlCl_3_.6H_2_O) was introduced to the mixture and left to rest for 5 min and 1 mL of NaOH was introduced. After that, the absorbance was measured at 506 nm using a UV‐spectrophotometer (Mashau, Ramatsetse, & Ramashia, 2021). The total flavonoid content was computed using the standard curve and reported as catechin (mg CE/g).

### 2.6 Determination of the proximate composition of the mutton

The moisture, ash, protein, fat, and fiber contents were determined through the procedures described in Association of Official Analytical Chemists AOAC (2007) using moisture content method number 945.32, ash content method number 923.03, protein method number 992.15, fat content method number 920.39, and crude fiber method number 985.33.

#### 2.6.1 Carbohydrates content

The carbohydrate amount was obtained by calculating the difference in percentage of moisture, fat, protein, ash, and crude fiber, using the following formula (Sultana, 2020):

(9) $$\text{Carbohydrates}\;\left(\%\right)=100-\left(\text{moisture}+\mathrm{fat}+\text{protein}+\text{crude fiber}\right)$$

### 2.7 Determination of the physical properties of mutton patties

#### 2.7.1 Cooking yield

Raw mutton patties were weighed, then cooked and surface‐dried with a filter and measured once more using an analytical scale balance (Ali et al., 2022). The cooking yield was calculated using the formula:

(10) $$\text{Cooking yield}=\frac{\text{Weight of cooked mutton patties}}{\text{Weight of}\;\mathrm{raw}\;\text{mutton patties}}\times 100$$

#### 2.7.2 Diameter reduction of patties during cooking

Diameter was determined following a method described by Kilincceker (2018) using the following formula:

(11) $$\begin{array}{c} \text{Diameter Reduction}\;\left(\%\right)=\\ \frac{\left(\text{Diameter of cooked}\;\mathrm{raw}\;\text{patties}-\text{Diameter of cooked patties}\right)}{\text{Diameter of}\;\mathrm{raw}\;\text{patties}}\times 100 \end{array}$$

#### 2.7.3 Color attributes

Color attributes were determined at three randomly chosen spots of the samples through a method outlined by Neethling et al. (2017) using Hunter Lab colorflex (Reston VA, U.S.A, D65). The instrument was initially calibrated using white and black tiles to determine the color parameters such as L* (lightness), a* (redness), and b* (yellowness). The whiteness index (WI), and yellowness index (YI) were determined using the equations (Mehdizadeh et al., 2020):

(12) $$\mathrm{Hue}=\tan^{-1}\;\left(b^{*}/a^{*}\right)$$

(13) $$\text{Chroma}={\left({a^{*}}^{2}+{b^{*}}^{2}\right)}^{1/2}$$

(14) $$\Delta E=\sqrt{{\left(\mathrm{\Delta L}\right)}^{2}+{\left(\mathrm{\Delta a}\right)}^{2}+{\left(\mathrm{\Delta b}\right)}^{2}})$$

(15) $$\mathrm{WI}=100-\sqrt{{\left(100-L^{*}\right)}^{2}+{\left(a\right)}^{2}+{\left(b\right)}^{2}}$$

(16) $$\mathrm{YI}=\frac{142.86b}{L^{*}}$$

#### 2.7.4 Texture profile analysis

**FIGURE 1:**
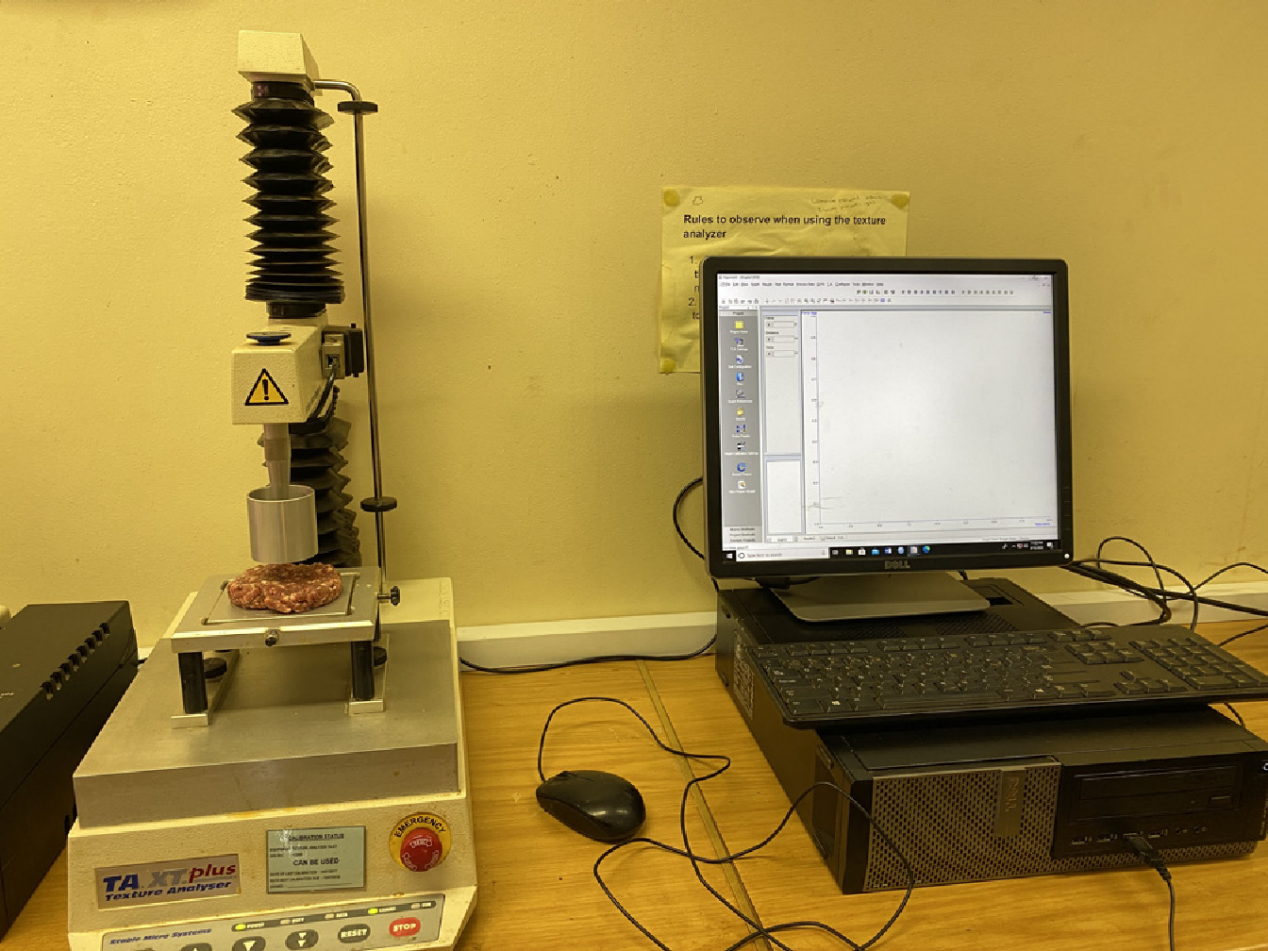
Texture analyzer to measure the hardness, springiness, cohesiveness, chewiness, and resilience of mutton patties.

The texture characteristics of grilled patties were determined using a method outlined by Andrés et al. (2011). The patties were pressed two times to 30% of their initial height amongst leveled plates. The texture analyzer (Stable Micro Systems, London, UK) (Figure 1) with a 75 mm diameter probe mms^−1^ (SMSP/75), linked up with a PC, and software provided by the texture operating system corporation (New York, United States) was used. Hardness (maximum power of the first compression phase, N), springiness (length of the discovered height of the sample on the succeeding (2nd) pressing split by the initial pressing length, mm/mm), cohesiveness (proportion of the opposite of negative areas of the number two cycle to an area of the first cycle, J/J), chewiness (hardness × cohesiveness × springiness, N), and resilience (area through the removal of the initial pressing split by the area of the initial pressing, J/J) were obtained.

### 2.8 Determination of lipid oxidation

Thiorbarbituric acid reactive substance (TBARS) values were obtained following the procedure described by Mashau, Munandi, and Ramashia (2021) for 21 days (0, 7, 14, and 21), where a standard solution of TBA was prepared in glacial acetic acid by dissolving 57.66 mg of TBA in 100 mL of glacial acetic acid. A TBA solution was prepared on each day of the analysis. One gram of each raw patties sample was put in a 25‐mL test tube with 50% glacial acetic acid (solvent). BHT (0.01%) was used to prevent the sample from oxidizing. The sample mixture was centrifuged (Rotina 380R‐Labotec Ecotherm, Midrand, South Africa) at 3000 rpm for 15 min and filtered into test tubes using Whatman papers. The mixture was boiled in a water bath at 95°C for 60 min, and test tubes were allowed to cool at ambient temperature (25 ± 2°C), and absorbance was read at 532 nm using a UV–visible spectrophotometer (Shimadzu UV‐1800, Japan).

### 2.9 Sensory analysis

Sensory evaluation was undertaken by 60 panelists with an age range of 18–50 years, encompassing university students and staff members who had previous knowledge of sensory analysis of meat. The formed patties were cooked by grilling in a Hobart CN85‐19 convection oven (Hobart Corp., Troy, Ohio, USA) for 20 min at 160°C to a core temperature of 80°C as measured at the geometrical center. To obtain homogeneous cooking, the patties were flipped over every 10 min. The prepared mutton patties were kept on warm trays on sealed plates for less than 10 min. Warmed portions (10 g) of each of the patties were placed on the white polystyrene plates and given to every participant with 3‐digit numbers in a random and consistent way for analysis. The panelists were given tap water to rinse their mouth after tasting every sample. Sensory testing was carried out in a well‐lit and well‐designed environment. To evaluate appearance, taste, texture, color, and total acceptability, analysis was undertaken using a 9‐point hedonic scale (9 = extremely like; 5 = indifferent; 1 = extremely dislike). All participants were given written information about the study and the products to be tasted before giving their informed consent.

### 2.10 Statistical analysis

The data obtained was analyzed by one‐way analysis of variance (ANOVA) using Statistical Package for the Social Sciences (SPSS) version 28 for Bambara groundnut flours. A two‐way analysis of variance was used for mutton patties. The disparity between the means of each analysis done in triplicates was determined using Duncan's multiple range test and the Ryan–Einot–Gabriel–Welsch (REGWQ) test for BGN flours and mutton patties, respectively. The acceptable significant difference was at the *p* ≤ .05 level. The spearman rank‐order correlation test was used to determine the correlation between cooking yield and diameter reduction.

## 3 RESULTS AND DISCUSSION

### 3.1 Bambara groundnut flour characteristics

The nutritional, functional, and physical properties of BGN flours are indicated in Table 1. The moisture content of red BGN flour (7.13%) was lower than that of cream BGN flour (8.85%), whereas brown BGN flour was not significantly different from cream and red BGN flours. However, the moisture content of all BGN flours was typically less than or between the anticipated range (0%–14%) in pulse flours (Buckman et al., 2018; Shanono & Muhammad, 2015; Skřivan et al., 2021). The low moisture content suggested that BGN flours would have an extended shelf‐life and be less susceptible to the proliferation of microorganisms during storage if they were packaged and stored appropriately (Mashau et al., 2022; Sruthi & Rao, 2021). Similarly, Oyeyinka et al. (2021) found higher moisture in cream BGN flour than in red and brown BGN flours. The ash content of all BGN flours was not significantly (*p* < .05) different from each other, with values varying from 2.89% to 3.39%.

**TABLE 1:** Bambara groundnut flour properties.

Properties	Cream	Red	Brown
Moisture (%)	8.85 ± 0.92^b^	7.13 ± 0.10^a^	8.17 ± 0.58^ab^
Ash (%)	2.89 ± 0.42^a^	3.14 ± 0.35^a^	3.39 ± 0.10^a^
Protein (%)	19.67 ± 0.21^a^	19.03 ± 0.70^a^	21.07 ± 0.98^b^
Fat (%)	6.67 ± 0.07^b^	6.70 ± 0.27^b^	6.42 ± 0.07^a^
Fiber (%)	4.76 ± 0.03^a^	7.73 ± 0.07^c^	5.89 ± 0.05^b^
Carbohydrates (%)	57.45 ± 0.11^c^	56.27 ± 0.08^b^	55.06 ± 0.47^a^
Energy (kcal/100 g)	368.48 ± 1.17^b^	361.50 ± 4.43^a^	362.29 ± 2.89^ab^
L*	88.07 ± 0.08^c^	81.82 ± 0.59^a^	83.43 ± 0.12^b^
a*	1.27 ± 0.02^a^	2.95 ± 0.08^b^	2.91 ± 0.05^b^
b*	10.48 ± 0.06^c^	7.18 ± 0.05^a^	8.39 ± 0.12^b^
Hue angle	82.78 ± 0.66^c^	67.01 ± 0.81^a^	70.79 ± 0.62^b^
Chroma	10.56 ± 0.06^c^	7.77 ± 0.07^a^	8.70 ± 0.34^b^
WI	84.07 ± 0.09^c^	80.23 ± 0.57^a^	81.20 ± 0.12^b^
YI	17.00 ± 0.10^c^	12.54 ± 0.18^a^	14.37 ± 0.21^b^
WAC (%)	182.49 ± 1.03^a^	204.38 ± 2.24^b^	201.91 ± 3.23^b^
OAC (%)	166.87 ± 1.11^a^	169.03 ± 3.72^ab^	174.21 ± 3.50^b^
EA (%)	49.64 ± 0.31^a^	51.73 ± 1.35^b^	52.83 ± 1.05^b^
ES (%)	51.37 ± 1.05^a^	49.32 ± 1.18^a^	51.26 ± 0.79^a^
BD (g/mL)	0.66 ± 0.01^a^	0.68 ± 0.01^b^	0.70 ± 0.00^c^
SC (g/g)	0.41 ± 0.03^b^	0.24 ± 0.05^a^	0.18 ± 0.13^a^
Dispensability (%)	57.33 ± 2.31^b^	52.00 ± 2.00^a^	48.00 ± 2.00^a^
Viscosity (cP)	2666 ± 5.57^a^	3402 ± 7.21^b^	3464.67 ± 4.51^b^
DPPH (%)	95.15 ± 3.67^b^	87.19 ± 0.87^a^	87.54 ± 0.74^a^
TPC (mg GAE/g)	0.79 ± 0.01^a^	0.98 ± 0.01^b^	0.91 ± 0.06^b^
TFC (mg CE/g)	0.06 ± 0.00^ab^	0.05 ± 0.00^a^	0.07 ± 0.01^b^

*Note*: Distinct small letters (superscripts) on the same row display that mean values are significantly different (*p* < .05).

Abbreviations: a, redness; b*, yellowness; BD, bulk density; BGN, Bambara groundnut; DPPH, 2,2 Diphenyl‐1‐pycryl‐hydrazyl; EA, emulsion activity; ES, emulsion stability; L*, lightness; OAC, oil absorption capacity; SC, swelling capacity; TFC, total flavonoids content; TPC, total phenolic content; WAC, water absorption capacity; WI, whiteness index; YI, yellowness index.

The protein content of brown BGN flour (21.07%) was significantly (*p* < .05) higher than that of cream (19.67%) and red BGN (19.03%) flours. Differences in the amount of protein in BGN flours might be due to variations in the type of the cultivar or unique genetic characteristics if cultivated in comparable conditions (Adeleke et al., 2018; Ikegwu et al., 2023). Oyeyinka et al. (2017) found significantly higher protein content in brown than red BGN flour. The high protein content makes BGN flour suitable for inclusion in protein‐rich diets or as a supplement for diets that lack adequate protein (Adeleke et al., 2017).

The fat content of brown BGN flour (6.42%) was less than that of cream (6.67%) and red BGN (6.70%) flours. The low‐fat content in BGN flours was anticipated given that BGNs are dry grains and typically have less than 10% fat when compared to oily grains like groundnuts and soybeans (Nwadi et al., 2020). Kaptso et al. (2015) stated that the low amount of fat in BGN flours might be because of their respective high protein content, as supported by the results in Table 1. A low amount of fat suggested that BGN flour could be preserved with minimal loss of quality until future use (Deepa & Umesh Hebbar, 2017).

The crude fiber of red BGN flour (7.73%) was greater than that of brown (5.89%), and cream BGN (4.76%) flours, respectively. Variations in the amount of crude fiber in BGN flours might be because of the type of cultivar (Ikegwu et al., 2023). Similarly, Shanono and Muhammad (2015) found a higher crude fiber content in red BGN flour than in brown BGN flour. Significant crude fiber content in BGN flours is important in reducing hypertension and serum cholesterol levels and providing protection from cardiovascular conditions. It also promotes healthy bowel function and increases fecal bulk (Aremu et al., 2022; Tan et al., 2021).

The carbohydrate content of cream BGN flour (57.45%) was higher than that of red (56.27%) and brown BGN (55.06%) flours. Regardless of the type of cultivar, carbohydrate was the most predominant component in all BGN flours. Adeleke et al. (2017) found higher carbohydrates in white (cream) BGN flour than in red BGN flour. Consuming BGNs can supply the body with enough energy due to their high carbohydrate content (Schulz & Slavin, 2021). The energy content of red BGN (361.50 kcal/100 g) flour was less than that of cream BGN (368.48 kcal/100 g) flour but not significantly different from brown BGN flour (362.29 kcal/100 g). Generally, the energy content of all BGN flours was high. The high energy content of cream BGN flour was due to its respective high carbohydrate (Bunmee et al., 2022).

The BGN flour's color properties are indicated in Table 1. The cream BGN (88.07) flour had a higher lightness (L*) value than the brown (83.43) and red BGN (81.82) flours. Therefore, BGN flours with lower L* values displayed significantly higher a* values, probably due to their relatively higher total phenolic content (Table 1), which is responsible for the red color (Bamshaiye et al., 2011; Parikh & Patel, 2018). The two BGN flour (red and brown) varieties had higher a* values than cream BGN flour. The higher the total phenolic content of the grain, the higher the a* value and lower the L* value (Tsamo et al., 2018). Oyeyinka et al. (2017) reported similar trends in the color of red, brown, and cream BGN flours, with respect to their phenolic content. The yellowness (b*) value of cream BGN (10.48) flour was higher than that of brown and red BGN flours which had values of 8.39 and 7.18, respectively. The higher b* value of the flour might be due to a greater amount of flavonoid content, as shown in Table 1, which is responsible for the yellow color of the grain (Khatun & Kim, 2021).

The chroma values of cream BGN flour were higher than those of brown and red BGN flours, varying from 7.77 to 10.56. The high chroma values of cream BGN flour were probably because of its relatively high b* value since it depends on it (Falade & Akeem, 2020; Ramashia et al., 2021). The higher chroma value of the flour displays the highly intense color of the sample, as perceived by consumers (Falade & Akeem, 2020; Mubaiwa et al., 2018). A similar trend of chroma and b* values was reported by Falade and Akeem (2020) in African mesquite bean powders. The hue angle of cream BGN flour was significantly higher (*p* < .05) than that of red and brown BGN flours.

The WI of cream BGN flour was higher than that of brown and red BGN flours, ranging from 80.23 to 84.07. The greater WI value of cream BGN flour was probably because of its relatively high L* value (Suliman et al., 2023). The higher WI value of cream BGN flour might also be due to its relatively lower phenolic content as well as genetic differences in the grain varieties (Mang et al., 2015; Uchechukwu‐Agua et al., 2015). Falade and Akeem (2020) found a higher WI of African mesquite bean flour due to its relatively high L* value. The YI of cream BGN flour was higher than that of brown and red BGN flours. The higher YI value of cream BGN flour was probably due to its correspondingly high b* value (Uchechukwu‐Agua et al., 2015). A comparable pattern of YI and b* was also reported by Uchechukwu‐Agua et al. (2015) in different cassava flour varieties.

The functional properties of BGN flours are indicated in Table 1. The WAC of brown and red BGN flours was higher than that of cream BGN flour, ranging from 182.49% to 204.38%. The greater polar amino acid residues of proteins that are water‐loving might be the reason for the higher WAC of brown and cream BGN flours (Adeleke et al., 2017; Arise et al., 2017). Thus, BGN flour with higher protein levels (Table 1) might have drawn up more water than those with a lower protein content (Marikkar et al., 2021). Iwe et al. (2016) stated that the disparity in WAC of various flours might be because of variations in their protein content, structural properties, and ability to absorb water. Furthermore, Adeleke et al. (2017) found higher WAC in brown than in white BGN flour.

The OAC of brown BGN flour was higher than that of red and cream BGN flours, with values varying from 166.89% to 174.21%. The disparity in the OAC of BGN flours might be due to the difference in the availability of non‐polar side chains that might have been attached to the hydrocarbon side chain of the oil amongst the BGN flours (Awuchi et al., 2019; Chandra et al., 2015).

The emulsion activity (EA) of cream BGN flour was lower than that of red and brown BGN flours. The lower EA of cream BGN flour might be because of its lower protein content in comparison to other BGN flours (Argel et al., 2020). In addition, proteins with higher solubility and lower surface hydrophobicity levels result in weaker interconnection surface covering lipid droplets (low EA) (Abiala et al., 2022; Argel et al., 2020). This result might also be due to the variety of the sample (Iwe et al., 2016). The EAs of BGN flours are higher than the EAs of cowpea, soya bean, and peanut (Abiala et al., 2022).

The bulk density (BD) of brown BGN flour was higher than that of red and cream BGN flours, with values varying from 0.66 to 0.70 g/mL. The disparity in BD might be because of the presence of starch content (Iwe et al., 2016); therefore, a large amount of carbohydrate might account for a greater amount of BD in BGN flours (Adeleke et al., 2017). The greater the BD, the smaller the packing volume and the stiffer the packaging material is needed, and vice versa (Awuchi et al., 2019; Mi & Ejeh, 2018). Adeleke et al. (2018) found higher BD in brown than white BGN flour. The BDs of BGN flours were comparable to the BDs of soya bean and cowpea flours (Abiala et al., 2022).

The swelling capacity (SC) of cream BGN flour was higher than that of red and brown BGN flours, varying from 0.18 to 0.41 g/g. Higher SC of cream BGN flour might be caused by high starch content, which is indicated by high carbohydrates (Table 1), particularly in starches that contain more branches of amylopectin (Iwe et al., 2016). The dispensability of cream BGN flour was higher than that of red and brown BGN flours. The high dispensability of BGN flours might be due to their relatively low WAC (Table 1) and higher gelling ability (Nzuta et al., 2022). Jenfa and Akinrinde (2019) found that the dispensability of BGN flour was less than that of the cowpea powder.

The viscosity of brown and red BGN was higher than that of cream BGN flours, ranging from 2666 to 3464.67 cP. The variation in viscosity may be caused by the intermolecular network's fragility, which might have led to the disintegration of BGN flour granules when gelatinized in hot water, resulting in a paste with a relatively low viscosity (Nzuta et al., 2022). Similarly, Falade and Nwajei (2015) reported higher viscosity in the brown variety than in the cream variety.

The antioxidant properties of BGN flours are indicated in Table 1. The DPPH content of cream BGN flour was significantly (*p* < .05) higher than brown and red BGN flours, varying from 87.19% to 95.15%. The high DPPH of cream BGN flour followed by red BGN flour might be due to their relatively high flavonoid content compared with brown BGN flour (Table 1) (Ahmed et al., 2015). Additionally, differences in BGN composition might have an impact on how hydrogen atoms were donated to DPPH, therefore affecting the DPPH content of BGN flour (Ramatsetse et al., 2023; Klompong & Benjakul, 2015). Oyeyinka et al. (2021) reported higher DPPH content in maroon BGN than in black and brown BGN flours with their respective high flavonoid contents. Nyau et al. (2015) reported a higher DPPH content in red BGN than in brown BGN flour.

The total phenolic content (TPC) of red and brown BGN flours was higher than that of cream BGN flour, ranging from 0.79 to 0.98 mg GAE/g. The difference in BGN coat colors might account for the disparity in TPC (Oyeyinka et al., 2021). Therefore, BGN grains that are darker in coat colors possess higher TPC than those with lighter coat colors (Adedayo et al., 2021; Parikh & Patel, 2018). Oyeyinka et al. (2017) found greater TPC in red BGN than in brown BGN flour. Tsamo et al. (2018) also reported higher TPC in red BGN than in brown and cream BGN flours, respectively. Therefore, consuming BGN grains may assist in fighting the negative effects caused by free radicals by functioning as free radical scavengers (Adedayo et al., 2021; Moyo et al., 2018). The total flavonoid content of brown and cream BGN flour was higher than that of red BGN flour, ranging from 0.05 to 0.07 mg/g CE. The differences in total flavonoid content of BGN flours might possibly be caused by differences in grain cultivars, assay procedures, and conditions of extracting (Ramatsetse et al., 2023).

### 3.2 Proximate composition of mutton patties

The proximate composition of raw mutton patties is shown in Table 2. The increase in percentage of BGN flours decreased the moisture content of patties compared to the control sample, ranging from 69.86% to 67.66%. The brown BGN variety significantly decreased the moisture content of patties compared with the cream and red BGN varieties, respectively. The reduction in the amount of moisture in formulated patties may be because of the low moisture content of BGN flours, as shown in Table 1. Correspondingly, Kasaiyan et al. (2023) observed a reduction in moisture content in lamb sausage due to the inclusion of chickpea powders.

**TABLE 2:** Proximate composition of mutton patties incorporated with different levels of Bambara groundnut flour.

Samples	Moisture (%)	Ash (%)	Protein (%)	Fat (%)	Fiber (%)	CHO (%)	Energy (kcal/100 g)
Control	69.86 ± 0.10^iD^	1.16 ± 0.05^aA^	19.64 ± 0.06^cC^	7.20 ± 0.02^eB^	0 ± 0.00^aA^	2.14 ± 0.11^aA^	151.92 ± 0.39^bcdefAB^
Brown 2.5%	69.35 ± 0.10^h^	1.45 ± 0.03^c^	19.57 ± 0.02^b^	7.11 ± 0.02^cd^	0.15 ± 0.01^bc^	2.37 ± 0.15^abc^	151.74 ± 0.42^bcde^
Brown 5%	69.11 ± 0.06^fg^	1.78 ± 0.00^de^	19.53 ± 0.01^ab^	7.06 ± 0.03^bc^	0.31 ± 0.01^def^	2.21 ± 0.08^ab^	150.50 ± 0.05^a^
Brown 7.5%	68.72 ± 0.23^d^	1.85 ± 0.01^f^	19.53 ± 0.01^ab^	6.94 ± 0.02^ab^	0.42 ± 0.01^fg^	2.49 ± 0.24^bcd^	150.95 ± 0.75^ab^
Brown 10%	68.25 ± 0.04^bc^	1.99 ± 0.00^g^	19.51 ± 0.02^a^	6.89 ± 0.12^a^	0.52 ± 0.01^gh^	2.79 ± 0.04^de^	151.68 ± 0.12^bcd^
Mean brown	68.86 ± 0.44^C^	1.77 ± 0.21^D^	19.54 ± 0.03^A^	7.02 ± 0.07^A^	0.35 ± 0.14^C^	2.47 ± 0.26^B^	151.22 ± 0.66^A^
Red 2.5%	69.19 ± 0.06^fgh^	1.41 ± 0.03^bc^	19.59 ± 0.02^bc^	7.12 ± 0.03^cd^	0.19 ± 0.00^bcd^	2.50 ± 0.08^bdc^	152.41 ± 0.42^cdefg^
Red 5%	68.77 ± 0.11^de^	1.77 ± 0.00^d^	19.56 ± 0.00^ab^	7.08 ± 0.04^cd^	0.40 ± 0.01^f^	2.43 ± 0.15^abc^	151.65 ± 0.33^bcd^
Red 7.5%	68.14 ± 0.03^b^	1.81 ± 0.01^ef^	19.55 ± 0.02^ab^	6.99 ± 0.03^ab^	0.55 ± 0.01^h^	2.95 ± 0.07^ef^	152.91 ± 0.12^fg^
Red 10%	67.66 ± 0.06^a^	1.98 ± 0.01^g^	19.54 ± 0.01^ab^	6.94 ± 0.02^a^	0.79 ± 0.18^i^	3.10 ± 0.18^f^	153.03 ± 0.80^g^
Mean red	68.44 ± 0.61^A^	1.74 ± 0.21^C^	19.56 ± 0.02^B^	7.03 ± 0.08^A^	0.48 ± 0.24^D^	2.74 ± 0.32^C^	152.50 ± 0.70^B^
Cream 2.5%	69.21 ± 0.10^gh^	1.38 ± 0.01^b^	19.56 ± 0.02^ab^	7.14 ± 0.03^de^	0.12 ± 0.00^b^	2.59 ± 0.06^cd^	152.84 ± 0.53^fg^
Cream 5%	68.98 ± 0.02^gh^	1.75 ± 0.00^d^	19.55 ± 0.02^ab^	7.08 ± 0.04^cd^	0.24 ± 0.01^cde^	2.39 ± 0.04^abc^	151.43 ± 0.20^abc^
Cream 7.5%	68.40 ± 0.02^c^	1.78 ± 0.01^de^	19.54 ± 0.01^ab^	6.98 ± 0.03^ab^	0.35 ± 0.01^ef^	2.95 ± 0.06^ef^	152.77 ± 0.09^efg^
Cream 10%	68.12 ± 0.02^b^	1.95 ± 0.01^g^	19.53 ± 0.04^ab^	6.92 ± 0.03^a^	0.42 ± 0.01^ef^	3.06 ± 0.03^ef^	152.68 ± 0.25^defg^
Mean cream	68.67 ± 0.46^B^	1.72 ± 0.21^B^	19.55 ± 0.02^AB^	7.03 ± 0.09^A^	0.28 ± 0.12^B^	2.75 ± 0.28^C^	152.45 ± 0.62^B^

*Note*: The values are expressed as the mean ± standard deviation. Means in the same column with distinct lowercases (superscripts) indicate a significant difference (*p* < .05). Brown, red, and cream (2.5%, 5%, 7.5%, and 10%) indicate different concentrations of different cultivars of Bambara groundnut flours, whereas means in the same column with distinct uppercases indicate a significant difference (*p* < .05) between the types of varieties.

Abbreviation: CHO, carbohydrates.

The ash content of formulated patties increased with the increase in percentage of BGN flours, varying from 1.16% to 1.99%. The brown BGN flour significantly increased the ash content compared with the red and cream BGN flours, respectively. The increased ash content of patties with BGN substitution might be due to the high amount of ash in BGN flours, as shown in Table 1. Similarly, Novello et al. (2019) reported an increase in the ash content of beef patties due to the inclusion of golden flaxseed powder. Furthermore, Alakali et al. (2010) found comparable data in beef patties added with BGN flour.

The protein content of formulated patties decreased with the increase in the percentage of BGN flours, ranging from 19.64% to 19.51%. The brown BGN flour decreased the protein content of the mutton patties more than the red BGN variety, respectively. The decrease in protein content of formulated patties might be due to the substitution of meat fat by BGN flours since meat fat holds a substantial amount of protein (Chandler & McSweeney, 2022). However, the low protein was still around the expected protein content (19%) of meat (Rama et al., 2019). Saikia et al. (2019) found a decrease in the protein content of duck meat patties as the amount of black gram powder increased. Jamaly et al. (2017) observed low protein content in beef meatballs due to the addition of wheat powder.

The fat content of formulated patties decreased with an increase in the percentage of BGN flours, with values ranging from 7.20% to 6.89%. Overall, all BGN varieties decreased the fat content of mutton patties equally. The control sample had a higher fat content of 7.20%. Gao et al. (2020) stated that the low‐fat content of meat patties might be due to the low fat content of legumes incorporated, as supported by the low‐fat content of BGN flours in Table 1. Morbos et al. (2019) found a decrease in the amount of fat in pork patties with an increase in the inclusion of mung bean powder.

The fiber content of formulated patties increased with an increase in the percentage of BGN flours compared to the control samples, varying from 0.00% to 3.10%. The red BGN variety increased the fiber content of the mutton patties more than the brown and cream BGN varieties, respectively. The improvement in the amount of fiber in formulated patties might be due to the amount of fiber content in BGN flours, as shown in Table 1. Similarly, Kahraman et al. (2023) found an increase in the amount of fiber in kirklareli meatballs incorporated with cowpea powder.

The carbohydrate content (CHO) of the control sample (2.14%) was less than that of the formulated samples, except for the sample added with 5% brown BGN flour. The red and cream BGN varieties increased the CHO content of the mutton patties more than the brown BGN variety. This was due to the high CHO content in BGN flours, as shown in Table 1. However, there was an inconsistent trend of CHO in patties with the inclusion of BGN flours because CHO content is calculated by the difference (Sousa et al., 2023). Therefore, samples with higher sums of proximate composition (protein, ash, moisture, and fiber) had lower CHO content, and vice versa. Embaby et al. (2016) found a similar trend in beef burgers added with lentil coat powder.

There was no consistent trend in the energy content of patties with the inclusion of BGN flours, with values ranging from 150.50 to 153.03 kcal/100 g. This is because the energy relies mostly on fat (9 kal/100 g), carbohydrate (4 kcal/100 g), and protein (4 kcal/100 g) (Kwon et al., 2020) and there was no effective difference in fat content; therefore, the sample with a higher sum of multiplication factors yielded a higher energy content. The red and cream BGN varieties improved the energy content of the patties more than the brown BGN variety. However, the control sample was not significantly (*p* < .05) different from samples formulated with brown 2.5% and 10%, and red 5% BGN flours. Öztürk‐Kerimoğlu et al. (2020) reported similar results in low‐fat sausages incorporated with quinoa flour.

### 3.3 Cooking yield and diameter reduction of mutton patties

The cooking yield and diameter reduction of mutton patties are indicated in Table 3. The cooking yield of mutton patties increased with an increase in the percentage of BGN flours, with values ranging from 76.39% for the control to 86.78% for patties added with a 10% cream flour variety. Overall, all BGN varieties increased the cooking yield of the mutton patties equally. The improvement in cooking yield in formulated patties might be due to the availability of dietary fiber, WAC, and OAC in BGN flours that absorbed or retained the fat and water throughout cooking, thereby forming a firmer component of the mutton patties matrix (Essa & Elsebaie, 2018; Mashau, Munandi, & Ramashia, 2021; Xu, 2017). Similarly, Chandler and McSweeney (2022) reported an increase in the cooking yield of chicken patties incorporated with yellow pea powder.

**TABLE 3:** The cooking yield and diameter reduction of mutton patties incorporated with different levels of Bambara groundnut flours and their correlation.

Samples	Cooking yield (%)	Diameter reduction (%)	*R*
Control	76.39 ± 0.03^aA^	17.73 ± 0.03^eB^	−.985
Brown 2.5%	81.28 ± 0.06^b^	16.49 ± 0.03^d^	
Brown 5%	83.21 ± 0.03^d^	15.54 ± 0.02^c^	
Brown 7.5%	84.03 ± 0.04^e^	15.04 ± 0.03^b^	
Brown 10%	86.75 ± 0.03^g^	13.99 ± 0.58^a^	
Mean brown	83.82 ± 2.27^B^	15.28 ± 0.94^A^	
Red 2.5%	81.39 ± 0.03^c^	16.25 ± 0.04^d^	
Red 5%	83.23 ± 0.02^d^	15.56 ± 0.04^c^	
Red 7.5%	84.12 ± 0.04^ef^	15.06 ± 0.02^b^	
Red 10%	86.80 ± 0.03^g^	13.94 ± 0.04^a^	
Mean red	83.89±2.25^B^	15.20±0.91^A^	
Cream 2.5%	81.31 ± 0.02^bc^	16.48 ± 0.02^d^	
Cream 5%	83.31 ± 0.03^d^	15.42 ± 0.03^bc^	
Cream 7.5%	84.12 ± 0.03^f^	15.10 ± 0.02^b^	
Cream 10%	86.78 ± 0.16^g^	13.89 ± 0.03^a^	
Mean cream	83.88 ± 2.27^B^	15.22 ± 0.97^A^	

*Note*: The values are expressed as the mean ± standard deviation. The mean in the same column with distinct lowercases (superscripts) indicates a significant difference (*p* < .05). Brown, red, and cream (2.5%, 5%, 7.5%, and 10%) indicate different concentrations of different cultivars of Bambara groundnut flours, whereas means in the same column with distinct uppercases indicate a significant difference (*p* < .05) between the types of varieties. *R* is the correlation coefficient of cooking yield and diameter reduction.

The increase in the percentage of BGN flours limited the diameter reduction of mutton patties, with values ranging from 17.73% to 13.89%. All BGN varieties decreased the diameter reduction of the mutton patties equally. The decrease in diameter reduction of formulated patties might be because of the greater water‐holding and swelling ability of BGN flours, as shown in Table 1 (Younis & Ahmad, 2018). Additionally, the decrease in diameter reduction might be attributed to the BGN flour's ability to bind, thereby keeping the meat components intact and preventing the patties dimensions from changing (Bunmee et al., 2022). The high diameter reduction value of the control sample was probably due to the fact that during cooking, the protein denatured, water evaporated, and fat drips led to weight loss and shrinkage of the patties (Chandler & McSweeney, 2022). Park et al. (2017) reported a decrease in diameter reduction in pork patties formulated with black rice powder. Furthermore, there was a negative correlation (*r* = −0.985) between the diameter reduction and the cooking yield of the patties. This was anticipated since BGN flour has the ability to absorb water, thereby improving cooking yield and limiting the diameter reduction of mutton patties during cooking.

### 3.4 Color properties of mutton patties

**FIGURE 2:**
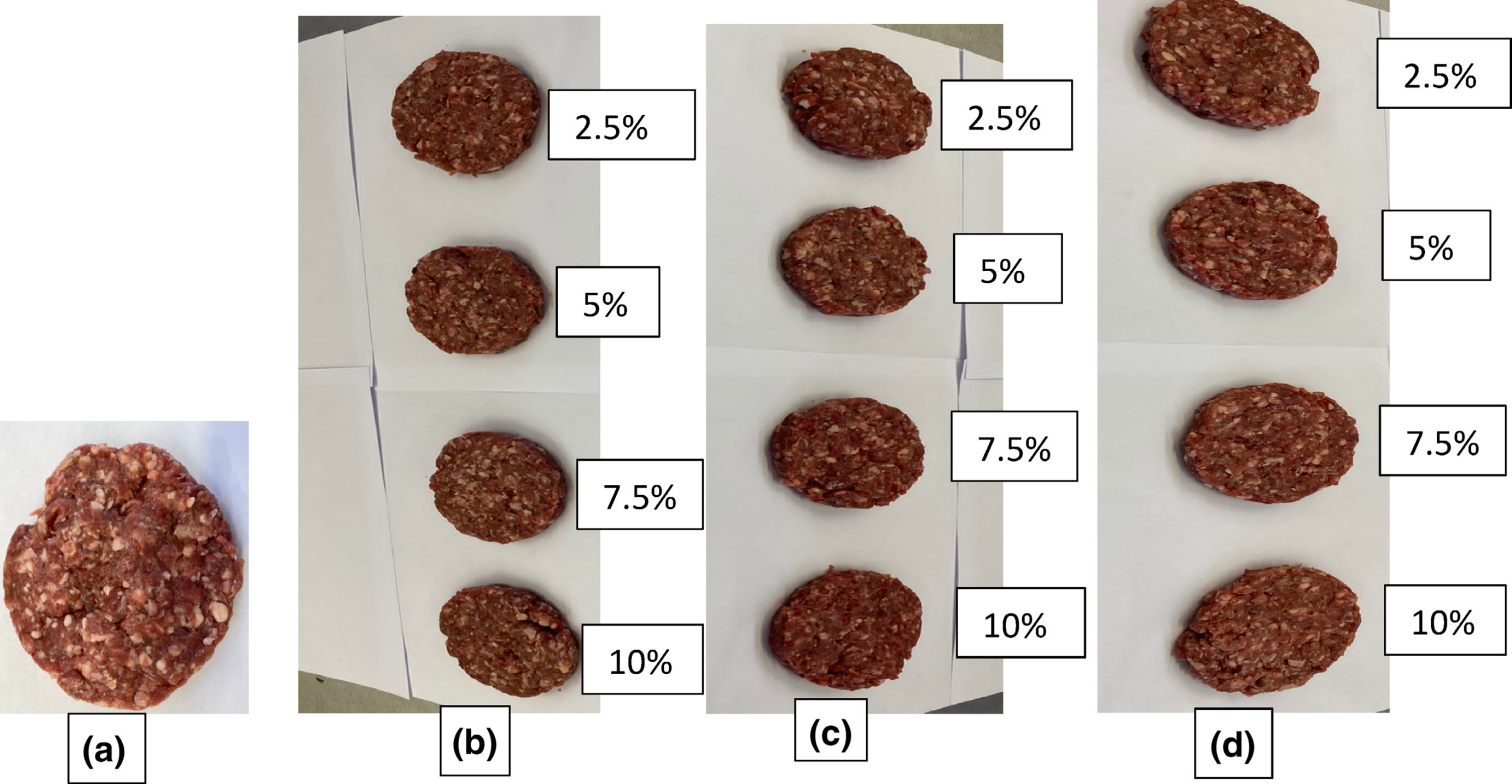
Mutton patties formulated with BGN flours (2.5%, 5%, 7.5%, and 10%). (a) 00% mutton patties; (b) mutton patties added with brown BGN flour; (c) mutton patties added with cream BGN flour; and (d) mutton patties added with red BGN flour. BGN = Bambara groundnut.

The color properties of raw mutton patties are depicted in Table 4, while Figure 2 shows images of mutton patties incorporated with different BGN flour varieties. The L* values of mutton patties increased with the addition of BGN flours, ranging from 54.04 to 58.59. The cream and brown BGN varieties increased the L* value of the patties more than the red BGN variety. However, patties added with 2.5% brown and red BGN flours did not differ from the control sample. The patties sample with the highest L* value was the one added with 10% cream BGN flour. The a* values of formulated patties decreased with the addition of BGN flours compared to the control. The cream BGN variety decreased the a* values of the patties more than the red and brown BGN varieties, respectively. Therefore, the improvement in L* values and reduction in a* values might be attributed to the dominant light color of BGN flours that diluted the red color of mutton patties, causing a reduction in meat myoglobin (Saikia et al., 2019). da Silva Felix et al. (2021) found an improvement in lightness and a reduction in redness in lamb patties with the inclusion of cassava flour. The b* values of mutton patties decreased with the inclusion of BGN flours, ranging from 18.38 to 15.49. All BGN varieties decreased the b* values of patties equally. However, patties added with 2.5% BGN flour did not significantly differ from the control sample. The decrease in b* values might be due to the low‐fat content of formulated patties, as shown in Table 2. Similarly, Salarkarimi et al. (2019) found a decrease in b* values of beef burgers incorporated with chestnut powder.

**TABLE 4:** Color properties of mutton patties incorporated with different levels of Bambara groundnut flour.

Samples	L*	a*	b*	Chroma	Hue angle	∆*E*	WI	YI
Control	54.04 ± 0.96^Aa^	14.90 ± 0.46^dC^	18.38 ± 0.43^dB^	23.66 ± 0.63^eB^	50.98 ± 0.24^aA^	0.00 ± 0.00^aA^	48.31 ± 1.14^aA^	48.61 ± 2.03^eB^
Brown 2.5%	55.47 ± 1.11^abc^	11.17 ± 1.35^bc^	17.68 ± 0.15^cd^	20.93 ± 0.86^cd^	57.79 ± 2.87^bc^	4.26 ± 1.51^bc^	50.79±0.73^bc^	45.54 ± 0.59^de^
Brown 5%	55.93 ± 1.67^abcd^	11.18 ± 1.43^bc^	16.72 ± 0.89^abc^	20.12 ± 1.53^bcd^	56.33 ± 1.98^b^	4.85 ± 1.42^bc^	51.52 ± 0.95^bcd^	42.70 ± 1.10^bcd^
Brown 7.5%	56.99 ± 0.10^bcde^	10.70 ± 0.45^bc^	16.30 ± 0.99^abc^	19.51 ± 0.63^abcd^	56.67 ± 2.60^b^	5.65 ± 1.10^bcd^	52.77 ± 0.32^de^	40.85 ± 2.53^abc^
Brown 10%	57.72 ± 0.37^cde^	9.97 ± 0.42^ab^	16.13 ± 0.68^abc^	18.96 ± 0.79^ab^	58.26 ± 0.29^bc^	6.57 ± 1.34^cd^	53.66 ± 0.02^ef^	39.91 ± 1.44^abc^
Brown mean	56.53 ± 1.27^B^	10.76 ± 1.02^B^	16.71 ± 0.90^A^	19.88 ± 1.15^A^	57.27 ± 2.03C	5.33 ± 1.46^BC^	52.18 ± 1.27^C^	42.25 ± 2.62^A^
Red 2.5%	54.96 ± 0.71^ab^	12.05 ± 0.39^c^	17.54 ± 0.45^cd^	21.29 ± 0.31^d^	55.50 ± 1.36^b^	3.17 ± 0.90^b^	50.38 ± 0.48^b^	45.61 ± 1.54^de^
Red 5%	55.07 ± 0.67^ab^	11.25 ± 0.64^bc^	16.42 ± 0.21^abc^	19.91 ± 0.54^bcd^	55.62 ± 1.14^b^	4.06±1.12^bc^	51.22 ± 0.58^bcd^	42.61 ± 0.05^bcd^
Red 7.5%	55.41 ± 0.41^abc^	10.82 ± 0.33^bc^	15.72 ± 0.10^ab^	19.08 ± 0.18^abc^	55.46 ± 0.90^b^	5.10 ± 0.90^bc^	51.34 ± 0.48^bcd^	40.52 ± 0.43^abc^
Red 10%	56.21 ± 0.66^abcde^	10.22 ± 0.29^abc^	15.26 ± 0.32^a^	18.37 ± 0.37^ab^	56.17 ± 0.28^b^	6.03 ± 0.37^cd^	52.16 ± 0.62^bcde^	38.78 ± 1.21^ab^
Red mean	55.21 ± 0.74^A^	11.09 ± 0.78^B^	16.24 ± 0.94^A^	19.66 ± 1.18^A^	55.69 ± 0.91B	4.59 ± 1.34^B^	51.27 ± 0.80^B^	41.88 ± 2.79^A^
Cream 2.5%	56.11 ± 0.42^abcd^	10.79 ± 0.77^bc^	17.03 ± 0.42^bcd^	20.17 ± 0.45^bcd^	57.67 ± 2.14^bc^	4.89 ± 0.30^bc^	51.74 ± 0.34^bcd^	43.36 ± 0.73^cd^
Cream 5%	56.65 ± 0.05^bcde^	10.24 ± 0.11^abc^	16.66 ± 0.35^abc^	19.55 ± 0.35^abcd^	58.41 ± 0.38^bc^	5.64 ± 0.85^bcd^	52.32 ± 0.21^cde^	42.01 ± 0.86^bcd^
Cream 7.5%	57.97 ± 1.03^de^	10.13 ± 0.17^ab^	16.37 ± 0.36^abc^	19.25 ± 0.34^abcd^	58.25 ± 0.64^bc^	6.57 ± 1.57^cd^	53.79 ± 1.00^ef^	40.34 ± 0.33^abc^
Cream 10%	58.59 ± 1.35^e^	8.79 ± 0.34^a^	15.49 ± 0.80^ab^	17.82 ± 0.71^a^	60.39 ± 1.63^c^	8.21 ± 1.76^d^	54.84 ± 1.83^f^	37.82 ± 2.79^a^
Cream mean	57.33 ± 1.28^B^	9.99 ± 0.85^A^	16.39 ± 0.74^A^	19.20 ± 0.99^A^	58.68 ± 1.60C	6.33 ± 1.69^C^	51.27 ± 0.80^D^	40.88 ± 1.21^A^

*Note*: Values are expressed as the mean ± standard deviation. Means in the same column with distinct lowercases (superscripts) indicate a significant difference (*p* < .05). Brown, red, and cream (2.5%, 5%, 7.5%, and 10%) indicate different concentrations of different cultivars of Bambara groundnut flours. Whereas means in the same column with distinct uppercases indicate a significant difference (*p* < .05) between the types of varieties.

Abbreviations: ∆*E*, color difference; a*, redness; b*, yellowness; L*, lightness; WI, whiteness index; YI, yellowness index.

The chroma of mutton patties decreased with an increase in the percentage of BGN flour, with values ranging from 23.66 to 17.82. All BGN varieties decreased the chroma values of patties equally. The reduction in chroma values might be attributed to the reduction in a* values of formulated mutton patties (Mashau, Munandi, & Ramashia, 2021). Thus, the inclusion of BGN flours did not boost the color intensity of patties since chroma decreased. Ball et al. (2021) found a reduction in chroma values in ground beef with the inclusion of pea protein. The hue angle of the control sample was lower than that of the formulated samples, ranging from 50.98 to 60.39^o^. The brown and cream BGN varieties increased the hue angle of patties more than the red BGN variety. The increase in hue angle was due to a decrease in a* values of formulated patties, as a higher hue angle represents lower redness of meat products (Ball et al., 2021). Furthermore, lower a* and chroma values with a high hue angle denote the browning of meat because of their strong correlation with metmyoglobin concentration (Khomola et al., 2021; Mashau, Ramatsetse, & Ramashia, 2021).

The color difference of formulated patties significantly increased with the addition of BGN flours, varying from 0.00 to 8.21. The cream BGN variety increased the color difference of patties compared with the brown and red varieties. The noticed disparities in the color of formulated mutton patties might be due to the presence of color pigments in BGN flours (Adewumi et al., 2022). Since the color differences of formulated patties are above 2, it indicates that they are noticeable (Mashau, Ramatsetse, & Ramashia, 2021; Moarefian et al., 2013). Similarly, Argel et al. (2020) found an increase in the color difference of low‐fat pork burgers with an increase in lentil flour concentration.

The WI of formulated patties increased with the inclusion of BGN flours, with values ranging from 48.31 to 54.84. Overall, the cream BGN variety decreased the WI of the patties more than the brown and red BGN varieties and control, respectively. The increase in WI might be due to an increase in L* values caused by the inclusion of BGN flours, which diluted the myoglobin in meat, thereby increasing WI (Saikia et al., 2019). Saricoban and Yilmaz (2010) found an improvement in the WI of beef meatballs with an increase in lightness. However, the YI of formulated patties decreased with the inclusion of BGN flours, ranging from 48.61 to 37.82. All BGN varieties decreased the YI of patties equally. The decrease in YI might be because of the reduction in b* values and fat content of the patties, as well as the dilution of myoglobin content (Kumar et al., 2020). A similar trend was reported by Saricoban and Yilmaz (2010) in beef meatballs.

### 3.5 Textural properties of mutton patties

**FIGURE 3:**
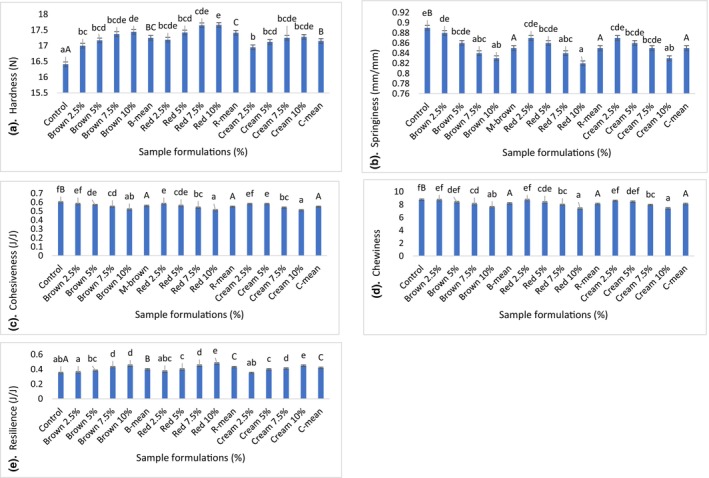
The texture properties of mutton patties incorporated with different levels of Bambara groundnut flour based on their colors. (a) Hardness, (b) Springiness, (c) cohesiveness, (d) Chewiness, and (e) Resilience, respectively. Means with distinct lowercases (superscripts) indicates significant difference (*p* < .05), whereas means in the same column with distinct uppercases indicate a significant difference (*p* < .05) in the type of the varieties.

The textural properties of raw mutton patties are indicated in Figure 3. The increase in the percentage of all BGN flours improved the hardness (a) and resilience (e) of patties compared to the control sample, ranging from 16.41 to 17.66 N and from 0.35 to 0.48 J/J, respectively. However, the resilience of the control was not different from the sample added with 2.5% brown and cream BGN flours. The red and brown BGN varieties increased the hardness of the patties more than the cream BGN variety, respectively. On the other hand, the red and cream BGN varieties increased the resilience of the patties more than the brown BGN variety. The significant increase in hardness and resilience might be due to the high WAC and dry matter of BGN flours (Table 1) that absorbed water in meat patties, thereby decreasing the moisture content of patties (Table 2), making them harder (Nuhriawangsa et al., 2023; Siddiq et al., 2009). Moreover, the improvement of dietary fiber due to the addition of BGN flours might have improved the water‐holding and ‐binding abilities of patties, thereby improving their hardness and resilience (Choi et al., 2010; López‐Vargas et al., 2014; Younis & Ahmad, 2018). Tahmasebi et al. (2016) found an increase in the hardness of sausages because of the inclusion of pigeon pea powder. Younis and Ahmad (2018) reported an increase in the hardness and resilience of buffalo patties with an increase in apple pomace flour. The addition of BGN flours decreased the springiness (b) of mutton patties, with values ranging from 0.89 to 0.82 mm/mm. All BGN varieties decreased the springiness of patties equally. The decrease in springiness might be due to the inclusion of BGN flours, which increased the fiber content of patties, thereby reducing the speed at which deformed patties spring back after initial compression (Hautrive et al., 2019).

The increase in the percentage of BGN flours reduced the cohesiveness (c) of patties compared to the control sample, ranging from 0.60 to 0.51 J/J. Overall, all BGN varieties decreased the cohesiveness of patties equally. The decrease in cohesiveness (c) of patties might be due to the inclusion of BGN flours, which led to a weakening of the inner bond structure of patties by reducing the stronger bonds formed between meat particles, resulting in a less dense and homogeneous structure of meat (Argel et al., 2022; Chandler & McSweeney, 2022). Similarly, Ghribi et al. (2018) reported a reduction in the cohesiveness of sausages incorporated with chickpea protein isolates. Verma et al. (2019) reported a reduction in the cohesiveness of goat nuggets due to the incorporation of amaranth seed powder.

The increase in the addition of BGN flours led to a decrease in the chewiness (d) of patties, with values varying from 8.77 to 7.38. However, there was no significant variation among the control and patties added with 2.5% brown and red BGN flours. All BGN varieties decreased the chewiness of patties equally. The decrease in chewiness might be due to the less difficult and more effective integration of BGN flours in the meat matrix (Sayas‐Barberá et al., 2021). Correspondingly, Öztürk‐Kerimoğlu et al. (2020) found a reduction in the chewiness of beef sausages with the incorporation of teff powder. Moreover, Sayas‐Barberá et al. (2021) found a reduction in the chewiness of beef patties with the incorporation of black quinoa powder.

### 3.6 Lipid oxidation of mutton patties

The thiobarbituric acid reactive substances (TBARS) values of raw mutton patties are indicated in Table 5. There was no significant difference in all samples on day zero. However, from Days 7 to 21, the TBARS values of the formulated mutton patties increased but were still less than those of the control sample within storage days, which had values varying from 0.08 to 0.88 mg MDA/kg. The red BGN variety delayed the lipid oxidation of the patties more effectively than the brown and cream BGN varieties. Mutton patties incorporated with 10% red BGN flour delayed lipid oxidation more effectively than the samples added with brown and cream BGN flours from Days 7 to 21. The low TBARS values of formulated patties within storage days compared to control might be due to the decrease in the amount of fat in formulated patties samples, as shown in Table 2, with the increase of BGN flours (Faid, 2019). Furthermore, low TBARS values might be because of the greater amount of polyphenolic compounds in BGN flours (Table 1), which act as free radical terminators and oxygen scavengers, thereby slowing down the process of lipid oxidation in mutton patties (Al‐Juhaimi et al., 2017; Bing et al., 2022).

**TABLE 5:** Lipid oxidation of mutton patties incorporated with different levels of Bambara groundnut flour (mg MDA/kg).

	Storage days			
Samples	0	7	14	21
Control	0.06 ± 0.00^a^	0.20 ± 0.00^g^	0.63 ± 0.00^p^	0.88 ± 0.00^w^
Control mean				0.44 ± 0.34^C^
Brown 2.5%	0.05 ± 0.00^a^	0.14 ± 0.00^f^	0.53 ± 0.01^mn^	0.77 ± 0.01^tu^
Brown 5%	0.06 ± 0.00^a^	0.12 ± 0.00^cdef^	0.49 ± 0.02^l^	0.76 ± 0.01^t^
Brown 7.5%	0.06 ± 0.00^a^	0.12 ± 0.00^cdef^	0.45 ± 0.02^j^	0.70 ± 0.01^r^
Brown 10%	0.05 ± 0.00^a^	0.10 ± 0.00^abc^	0.39 ± 0.02^i^	0.60 ± 0.00^o^
Brown mean				0.38 ± 0.27^B^
Red 2.5%	0.05 ± 0.00^a^	0.13 ± 0.00^ef^	0.51 ± 0.01^lm^	0.78 ± 0.02^u^
Red 5%	0.05 ± 0.00^a^	0.12 ± 0.00^cdef^	0.50 ± 0.01^l^	0.74 ± 0.01^s^
Red 7.5%	0.05 ± 0.00^a^	0.11 ± 0.00^cde^	0.44 ± 0.02^j^	0.65 ± 0.02^q^
Red 10%	0.06 ± 0.00^a^	0.08 ± 0.01^b^	0.35 ± 0.02^h^	0.54 ± 0.01^n^
Red mean				0.36 ± 0.26^A^
Cream 2.5%	0.05 ± 0.00^a^	0.14 ± 0.00^f^	0.54 ± 0.01^n^	0.79 ± 0.01^v^
Cream 5%	0.06 ± 0.00^a^	0.13 ± 0.00^ef^	0.50 ± 0.01^l^	0.77 ± 0.00^tu^
Cream 7.5%	0.05 ± 0.00^a^	0.11 ± 0.00^cde^	0.47 ± 0.01^k^	0.70 ± 0.01^r^
Cream 10%	0.06 ± 0.00^a^	0.10 ± 0.00^abc^	0.40 ± 0.02^i^	0.63 ± 0.00^p^
Cream mean				0.38 ± 0.27^B^

*Note*: Values are expressed as the mean ± standard deviation. Means in the same column with distinct lowercases (superscripts) indicate a significant difference (*p* < .05) within that day. Means in the same row with distinct lowercases (superscripts) indicate a significant difference (*p* < .05). Brown, red, and cream (2.5%, 5%, 7.5%, and 10%) indicate different concentrations of different cultivars of Bambara groundnut flours, whereas means in the same column with distinct uppercases indicate a significant difference (*p* < .05) between the types of varieties.

Abbreviation: TBARS, thiobarbituric acid reactive substances.

The significant increase in TBARS values during the storage period might influence the oxidation of double bonds of polyunsaturated fatty acids as well as the production of volatile compounds in meat (Dilnawaz et al., 2017; Mashau, Ramatsetse, & Ramashia, 2021). Although TBARS values increased in all samples during the storage days, the values were still within the permissible limits of less than 1 MDA/kg (Cerón‐Guevara et al., 2020; Dilnawaz et al., 2017). Comparable data were found by Dilnawaz et al. (2017) in mutton blocks incorporated with green coffee bean powder. Furthermore, Alakali et al. (2010) found a delay in lipid oxidation of beef patties incorporated with BGN flour.

### 3.7 Sensory properties of mutton patties

The sensory properties of mutton patties formulated with BGN flours are indicated in Table 6. There was no noticeable difference in the appearance, aroma, texture, and overall acceptability scores of the formulated patties compared to the control sample. Furthermore, there was no noticeable difference in the taste of the formulated patties (except for 10% brown and 7.5% cream BGN flours) compared to the control sample. The sensory attributes liked by panelists for formulated patties might be due to high OAC of BGN flours, as shown in Table 1, which improves the sensation of chewing, the taste, and preserves the flavor of meat (Adewumi et al., 2022; Arise et al., 2019). The taste scores of mutton patties samples formulated with 10% brown BGN flour (6.45) and 7.5% cream BGN flour (6.27) were less than those of the control sample. The lower taste score might be due to the stronger flavor of BGNs, which panelists disliked; this dominated the flavor of formulated mutton patties (Motamedi et al., 2015; Novikasari et al., 2020). Nevertheless, the sensory properties of all samples scored above the acceptable level of 5 on the 9‐hedonic scale. Therefore, the findings of the study imply that BGN flours may be used as a fat substitute in low‐fat mutton patties without causing drastic changes in the sensory attributes of the meat. Similar results were reported by Argel et al. (2020) in low‐fat pork patties incorporated with lentil powder. Furthermore, Alakali et al. (2010) found comparable results in beef patties incorporated with 5% BGN powder.

**TABLE 6:** Sensory properties of mutton patties incorporated with different levels of Bambara groundnut flour.

Samples	Appearance	Aroma	Taste	Texture	Overall acceptability
Control	7.05 ± 1.61^abc^	6.66 ± 1.83^ab^	7.53 ± 1.93^b^	7.23 ± 1.31^a^	7.31 ± 1.62^a^
Brown 2.5%	7.60 ± 1.42^abc^	7.02 ± 1.56^ab^	7.55 ± 1.31^b^	7.45 ± 1.48^a^	7.47 ± 1.32^a^
Brown 5%	7.06 ± 1.84^abc^	6.85 ± 1.49^ab^	6.87 ± 1.42^ab^	6.98 ± 1.73^a^	6.81 ± 1.77^a^
Brown 7.5%	6.79 ± 1.99^ab^	6.63 ± 1.67^ab^	6.79 ± 1.85^ab^	6.87 ± 1.65^a^	6.74 ± 1.86^a^
Brown 10%	6.92 ± 1.86^c^	6.77 ± 1.82^ab^	6.45 ± 2.23^a^	6.81 ± 2.02^a^	6.76 ± 1.84^a^
Red 2.5%	6.87 ± 1.48^abc^	6.76 ± 1.75^ab^	6.79 ± 1.77^ab^	6.97 ± 1.64^a^	7.05 ± 1.47^a^
Red 5%	7.46 ± 1.32^ab^	7.23 ± 1.56^b^	6.95 ± 1.83^ab^	7.28 ± 1.40^a^	7.25 ± 1.52^a^
Red 7.5%	7.24 ± 1.74^abc^	6.73 ± 1.71^ab^	6.79 ± 2.11^ab^	7.23 ± 1.48^a^	7.00 ± 1.82^a^
Red 10%	6.89 ± 1.96^abc^	7.03 ± 1.85^ab^	6.77 ± 2.20^ab^	7.13 ± 152^a^	6.76 ± 2.13^a^
Cream 2.5%	7.18 ± 1.82^abc^	6.94 ± 1.88^ab^	7.03 ± 2.40^ab^	7.19 ± 1.84^a^	7.02 ± 1.92^a^
Cream 5%	6.61 ± 2.26^a^	6.81 ± 1.61^ab^	6.77 ± 2.15^ab^	6.95 ± 1.87^a^	6.90 ± 1.91^a^
Cream 7.5%	7.00 ± 1.70^abc^	6.40 ± 2.04^a^	6.27 ± 2.35^a^	7.00 ± 1.98^a^	6.73 ± 2.09^a^
Cream 10%	7.29 ± 1.63^abc^	6.90 ± 1.58^ab^	6.79 ± 2.07^ab^	7.18 ± 1.47^a^	7.03 ± 1.75^a^

*Note*: Values are expressed as the mean ± standard deviation. The mean in the same column with distinct lowercases (superscripts) indicates a significant difference (*p* < .05). Brown, red, and cream (2.5%, 5%, 7.5%, and 10%) indicate different concentrations of different cultivars of Bambara groundnut flours.

## 4 CONCLUSIONS

The inclusion of BGN flour varieties in mutton patties can be utilized as a natural preservative, fat substitute, ash, and fiber booster. The increase in percentages of BGN flours decreased the protein content of mutton patties, but it was still within the expected protein content (19%) of meat. The cooking yield and diameter reduction of mutton patties were also improved due to the increase in the percentage of BGN flours up to 10%. Furthermore, the inclusion of BGN flour from 2.5 to 10% was successful in delaying the lipid oxidation, indicating that it may be used to improve the shelf‐life of mutton patties because of its antioxidant properties. The sensory properties (appearance, taste, texture, color, and overall acceptability) of formulated patties were not significantly different from the control sample; however, they were above the acceptable score of 5. All BGN varieties had positive effects on the mutton patties, especially the red BGN variety, followed by the brown and cream BGN varieties, respectively. Furthermore, the inclusion of 10% red BGN flour in mutton patties is highly recommended since it retains the color, reduces the moisture content, delays lipid oxidation, increases the ash, fiber, carbohydrates, and energy contents, as well as hardness and resilience, compared with other BGN varieties. The results of the study show that Bambara groundnut can be used as an additive in mutton patties without detrimental effects on the quality parameters.

## AUTHOR CONTRIBUTIONS

**Kgaogelo Edwin Ramatsetse:** Data curation (equal); formal analysis (equal); funding acquisition (lead); investigation (equal); methodology (equal); validation (equal); writing – original draft (lead). **Shonisani Eugenia Ramashia:** Project administration (lead); resources (equal); supervision (equal); writing – review and editing (equal). **Mpho Edward Mashau:** Conceptualization (lead); funding acquisition (supporting); project administration (equal); supervision (equal); validation (equal); writing – review and editing (equal).

## FUNDING INFORMATION

This work was supported as part of MSc (Food Science and Technology) by the National Department of Agriculture, Land Reform and Rural Development.

## CONFLICT OF INTEREST STATEMENT

The authors have no conflict of interest to declare.

## ETHICS STATEMENT

This study does not involve any human or animal testing.

## CONSENT FOR PUBLICATION

All listed authors have read the final manuscript and provided consent for publication.

## Data Availability

The data that support the findings of this study are available on request from the corresponding author.
